# Leveraging African Ancestry Diversity for Global Advances in Alzheimer Disease Genetics

**DOI:** 10.1002/alz70855_105506

**Published:** 2025-12-24

**Authors:** Farid Rajabli, African Dementia Consortium, Alzheimer's Disease Sequencing Project (ADSP), Anthony J Griswold

**Affiliations:** ^1^ Dr. John T. Macdonald Foundation Department of Human Genetics, University of Miami Miller School of Medicine, Miami, FL, USA; ^2^ John P. Hussman Institute for Human Genomics, University of Miami Miller School of Medicine, Miami, FL, USA; ^3^ College of Medicine, University of Ibadan, Ibadan, Oyo, Nigeria; ^4^ University of Pennsylvania, Philadelphia, PA, USA

## Abstract

Alzheimer disease (AD) genetic research increasingly recognizes the importance of ancestral diversity in understanding the genetic mechanism of AD. The “Recruitment and Retention for Alzheimer's Disease Diversity Genetic Cohorts in the ADSP (READD‐ADSP)” initiative addresses this need by building a resource to study AD genetics across diverse populations in the United States and Africa. This global collaboration, spanning four U.S. sites and ten African countries through the Africa Dementia Consortium, aims to examine and understand genetic and non‐genetic factors involved in AD risk and progression.

READD‐ADSP employs genomic and epidemiological methods to investigate AD across diverse populations in the United States and Africa. This international collaboration integrates whole‐genome sequencing and blood biomarker data, along with social determinants of health (SDOH) and clinical measures, to examine the interplay among genetic, ancestral, and SDOH factors. Ancestral analyses define population structure, assess risk‐gene effect heterogeneity (e.g., APOE), and explore locus‐specific variation (e.g., ABCA7). Through comprehensive association analyses, the study aims to evaluate the generalizability of known AD risk loci to African ancestry populations and identify novel genetic risk or protective factors.

Preliminary analyses reveal substantial ancestral variation within African populations, particularly when comparing West and East African populations. Previous AD genetic studies showed heterogeneity in risk effect size such as the *APOE4*allele and risk marker heterogeneity at the same risk locus across ancestries such as *ABCA7* gene, but these differences remain understudied in African continental populations. Our findings highlight the potential of diverse genomic backgrounds to uncover protective genetic factors, such as the African‐specific protective *PSG2* locus, found to confer a protective effect among *APOE4* carriers. By incorporating underrepresented groups, this research can broaden our understanding of AD and inform interventions that benefit globally.

The study of genetic variations within African and African American populations opens new avenues for identifying novel risk and protective AD loci and refining our understanding of AD etiology. Future work will benefit from continued cross‐continental collaboration integrating genetic data with SDOH factors. The READD‐ADSP initiative will enhance our understanding of AD, informing precision therapies that are globally tailored to the unique genetic profiles of all populations.

